# Finding common ground: collaboration to solve ‘wicked’ problems

**DOI:** 10.1017/S0266462325100378

**Published:** 2025-07-29

**Authors:** Linda Mundy, Guy Maddern

**Affiliations:** 1 School of Public Health, Faculty of Health and Medical Sciences, University of Adelaide, Adelaide, SA, Australia; 2 Chair, HTAi Asia Policy Forum, University of Adelaide, Adelaide, SA, Australia; 3 Discipline of Surgery, The Queen Elizabeth Hospital, Adelaide, SA, Australia

**Keywords:** collaboration, multistakeholder, health technology assessment, decision making, health policy, Asia

## Abstract

Collaboration is both a process and an outcome. Collaboration is based on the idea that interactions between participants with a common goal, working together as partnerships and sharing resources, can solve complex or “wicked” problems that are not possible to solve in isolation. Collaboration may be simple, occurring between individuals, or more complex interorganizational arrangements across sectors, with the life cycle and size of the collaboration determined by the issue at hand. HTA collaborations may involve a wide range of stakeholders, including HTA agencies at the national, regional, or global level, academia, government (including regulatory authorities), industry, clinicians, providers, and patient organizations. Regardless of the number or type of participants, collaborations need a shared understanding of the common goal, an agreement on aims, and a commitment to shared solutions.

Industry and agency members of the Health Technology Assessment International (HTAi) Asia Policy Forum (APF) met in Seoul, South Korea, in November 2024 for open discussions on how to facilitate and improve the collaborative process between all stakeholders in the health system, including government, HTA agencies, industry, academia, clinicians, as well as patients. Over the three days, these discussions identified some of the risks and obstacles to collaboration in the region, how to develop and use collaboration better, as well as articulating the value and benefits of collaboration both in the region and globally.

## Introduction

Each year, members of the Health Technology Assessment International (HTAi) Asia Policy Forum (APF) attend a three-day meeting to discuss an HTA issue of concern, with discussions informed by a background paper (1). The 2024 APF was attended by twenty-three representatives from thirteen not-for-profit organizations (HTA agencies, payers, and health systems) and twenty-six representatives from thirteen for-profit organizations (pharmaceutical, biotech, and medical device companies) from around the Asia region. As the APF is designed to promote open and constructive dialogue, without fear or favor, these meetings are conducted under the Chatham House Rule, in which participants are free to share information obtained during the meeting, but the identity or affiliation of the person providing the information cannot be revealed (2). Open discussions during the 2024 APF identified some of the risks and obstacles to regional collaboration, proposed ways to improve engagement and strengthen partnerships, and highlighted the value and benefits of collaboration both regionally and globally. This paper is the author’s summary of some of the key messages and discussions of the 2024 APF.

### The fundamentals of collaboration

Collaboration, defined as “*working together with others to achieve a common goal”* (3), can be both a process and an outcome (4). Collaboration is based on the idea that interactions between participants with a common goal, working together and sharing resources, can solve complex or “wicked” problems, such as the recent COVID-19 pandemic, that are not possible to solve in isolation with individual capacity. There is, however, no “recipe” for the ideal collaboration. Collaborations in HTA may be simple, occurring agency to agency between individuals, or more complex arrangements across one or more sectors, involving a wide range of stakeholders, including academia, government, regulatory authorities, industry, clinicians, providers, or patient organizations (5).

The life cycle and size of a collaboration, as well as the level of governance and management required, will be determined by the issue trying to be solved. Collaborative projects can range from short-term, time-limited projects with a small number of partners that target a specific issue with prespecified outcomes, such as the development of an action plan for rare diseases (6). More permanent multi-stakeholder collaborations require a long-term commitment from all partners and may rely heavily on one committed partner to champion and coordinate the project, such as Project Orbis, a global collaborative regulatory review program led by the United States Food and Drug Administration (FDA) (7–9). Regardless of scope, a successful collaboration requires participants to have a shared understanding of the common “problem” or goal, agree on collaborative aims and commit to shared solutions (10;11), with clear communication between members being key, especially when negotiating or assigning roles and responsibilities (4).

Although cooperation, communication, and contribution are fundamental pillars of successful collaboration, trust and commitment, both of which can be challenging and time-consuming, are critical to building, developing, and maintaining collaborative projects (12). Trust can be built by sharing resources and information as well as demonstrating competency and good intentions. Successful collaborations based on trust create confidence in the collaboration and, in so doing, build more trust (13;14). Commitment demonstrates the belief of participants that collaborative relationships are important enough to warrant the effort to maintain the project through its lifespan (15). Instilling confidence in the organizational competence and expected performance of the project will create commitment; however, failing to fully commit may undermine trust in the relationship, leading to, at best, “collaborative inertia” or, at worst, project failure (10).

### The HTAi Asia Policy Forum meeting

**Figure 1.**
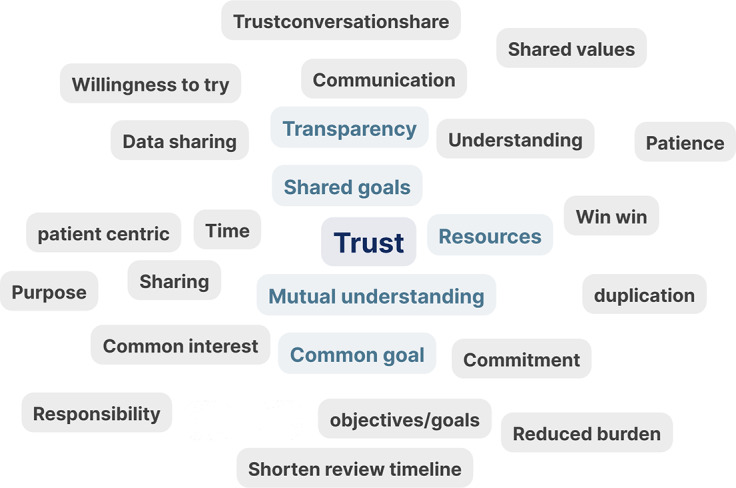
Word cloud generated by APF delegates, defining collaboration on Day 1.

Before discussions got underway in earnest, delegates were asked in a premeeting poll to describe the necessary ingredients for a successful HTA-based collaboration. The resulting word cloud reiterated the importance of the values discussed above (Figure 1), showing that “*Trust”* was overwhelmingly the key factor identified, followed by *‘Transparency,” ‘Shared goals”* and *‘Mutual understanding.”*

The structure of the 2024 APF consisted of a plenary delivered by the host agency, followed by panel sessions highlighting ongoing collaborative projects in the region. For the final session on Day 2, an equal number of for-profit and not-for-profit delegates were assigned to breakout groups to discuss predefined topics, including facilitators and mechanisms of collaboration, challenges and obstacles to collaboration, and the types of collaboration considered of greatest importance in the Asia region.

### Facilitators and barriers to collaboration

The literature discusses numerous potential benefits of collaboration, including building relationships, creating connections, and streamlining work processes to deliver efficiencies. Collaboration can deliver increased capacity and capability when partners exchange information and skills, learning from each other. Collaboration can be facilitated by shared decision-making and recognizing partners” values and goals, which may take time and effort, especially when multiple stakeholders with differing perspectives are involved (4).

Breakout group discussions corroborated many of these points, with delegates agreeing that whilst there are many facilitators of collaboration, some of these can easily become barriers:

- Shared challenges and even crises with a shared goal offer opportunities for collaboration and may result in better outcomes than individual efforts, as demonstrated by the recent COVID-19 pandemic.
- Effective communication is key. It is incredibly helpful to be able to talk in the same language, especially when trying to understand the local context.
- Group members identified that the development of trust with and between collaborative partners is an important facilitator; however, it was agreed that this may prove difficult with time-limited projects. Developing clear guidelines for government-industry collaborations to standardize transparency practices or using independent entities or mediators to act as bridges between stakeholders may improve or facilitate trust between partners.
- Having a common platform was also viewed as valuable, with forums such as the APF or the regional network, HTAsiaLink, providing a “safe” space where stakeholders can come together and share information. Connections made in forums such as these can lead to a “force multiplier effect” – connecting with the right people, leading to further collaborative opportunities.
- The need for adequate financial and political support was also discussed. Funding was viewed as both a potential facilitator and a barrier to collaboration, where the success or failure of a collaborative project may depend on sufficient funding to provide the right resources, workforce, infrastructure, and administration. Conversely, insufficient funding may precipitate the end of a collaboration, as in the case of many time-limited collaborations funded by governments. Political will was identified as an essential facilitator and a potential barrier that should not be underestimated, as collaborations may be brought to a premature end with a change in government or policy direction.

As stated in the literature, collaboration is not always easy and does not always work. Collaborative partners may find that the goals or culture of the collaboration differ from their own, creating conflict. Some partners in the collaboration may find it difficult to build trust in the relationship, whilst others are resistant to working together from the outset. It is important to identify leaders within the collaboration at the outset who can facilitate debate whilst allowing the healthy expression of different perspectives, which may assist in resolving any conflicts that may arise (4). Collaboration can also be stifled by a top-down administration intent on maintaining a hierarchy over effectiveness. Other organizational barriers include time pressure, competing priorities, operational constraints or a lack of leadership and support (16).

Breakout Group One agreed with the literature that it is important to find the right collaborative mechanism, starting with understanding each partner’s needs, and recognizing how all partners can benefit and become stronger by collaborating, creating a “win-win” situation. When setting up a new collaboration, the rules of engagement should be defined up front, along with clear governance and processes. The early engagement and involvement of stakeholders to contribute to the “codesign” of the collaboration will create a sense of ownership for all stakeholders and help to create a safe space for the collaboration to operate in. Finally, all partners need to be willing to learn in the process, to be flexible and adaptive, and to take the time to reflect on the process.

The barriers to collaboration may be more subtle than a lack of funding or political will. Care must be taken so that dominant partners do not overwhelm or ignore differences in the capacity of other, less well-resourced parties to participate in the collaborative process. Respect for all partners demonstrates goodwill and empowers participants, especially by considering any language or cultural differences in the group. Power imbalances within the collaboration may lead to a “what’s in it for me?” mentality, with resource-rich partners resenting partners with fewer resources or capacity, viewing them as “passengers,” accruing benefits without contributing resources or failing to implement planned actions (15). Setting clear guidelines around communication and expectations at the outset of a collaboration may negate some of these issues (4).

Breakout Group Two discussed some of the obstacles to collaboration in the Asia region, and considered some potential solutions:

- Firstly, a lack of trust and transparency between stakeholders, such as government and industry, was identified as a potential obstacle. Governments often face limitations in transparency, which breeds distrust. Concerns were expressed over government-funded initiatives such as sandbox programs, where a sense of bias can be perceived with the endorsement of specific products. The Group felt that clear guidelines to standardize transparency practices should be developed for sensitive collaborations such as those between government and industry. Transparency may be better achieved by using independent entities or mediators to act as a bridge between stakeholders to facilitate trust.
- Poor communication, misalignment of goals, and differing expectations among stakeholders were identified as barriers to successful collaboration, especially during time-limited, complex projects where there is limited capacity to engage in consistent communication. Group Two felt that clearly defining and aligning shared goals and expectations at the outset would ensure all parties understood the project’s purpose. In addition, flexible project management methods allow time to build alignment to better accommodate each stakeholder’s priorities.
- Leadership and direction can act as a facilitator; however, insufficient high-level support and leadership can also be a barrier by slowing down or even halting projects. Different leadership perspectives (e.g., national versus organizational priorities) may also lead to inconsistent levels of support for collaborative initiatives. The use of persuasive communication could help showcase the value of collaboration to leaders, enabling them to recognize the long-term benefits to the health system. In addition, the creation of a high-level consensus-building strategy could help to secure an early “green light” from the leadership group and avoid wasting time and resources.
- Regulatory and structural barriers can be major obstacles to collaborative efforts with complex or rigid regulations, making it difficult to align processes or gain timely approvals for collaborative projects. In addition, a lack of infrastructure and support mechanisms, including standardized frameworks, may add complexity to projects. These hurdles may be overcome by streamlining regulatory processes or developing frameworks that allow for expedited approvals, especially for initiatives with clear public health benefits. This was highlighted in the APF plenary describing the collaborative relationship between South Korea’s HTA agency, the National Evidence-based Healthcare Collaborating Agency (NECA), and the Health Insurance Review and Assessment Service (HIRA). This relatively new integrated review and assessment collaborative process for innovative medical devices is expected to reduce the evaluation period from 390 to 80 days, with regulatory approval, HIRA’s reimbursement decision-making, and NECA’s HTA all taking place simultaneously. For another example, see the APF 2024 background paper (1) for a discussion on Project Orbis, a global collaborative regulatory review program led by the FDA to promote efficiency through early engagement between regulatory bodies and manufacturers, facilitating faster patient access to innovative cancer therapies. Although one country in the region, Singapore, is a member of this collaboration, the APF briefly discussed the need for a similar project in the region.
- A difference in appetite for risk or level of tolerance of risk among stakeholders was viewed as a potential obstacle, with some stakeholders opting for being conservative, whilst others advocate for an innovative approach. These opposing views may be harmonized by conducting a joint risk assessment (e.g., a SWOT or feasibility analysis) to create a balanced understanding of all the risks and benefits of a project. Others in Group Two advocated embracing a “learn by doing” approach, especially in low-risk settings, to assist all parties to incrementally adapt to different levels of risk, an approach that has been successfully implemented at the national level by China’s National Reimbursement Drug List.
- An important collaborative obstacle to consider in the region is the presence of organizational biases and cultural differences, including one stakeholder potentially dominating the collaboration, which may limit objective decision-making and cooperation. Promoting a collaborative culture that creates a fair decision-making process, prioritizing shared benefits, and minimizing individual biases may help negate these biases. It may also be useful to encourage each party to view collaboration as a way of enhancing health system outcomes rather than for personal or organizational gains.

There were many take-home messages from the Breakout Groups, including, above all else, building trust and transparency will lay the foundation for a successful collaboration, encourage all stakeholders to voice their thoughts and participate in the HTA process, start small, and a learn-by-doing approach empowers all partners.

### Case studies

Many collaborative frameworks, including discussions at HTAi’s 2024 Global Policy Forum, consider a checklist of elements that should be identified at the outset of a collaboration to increase the likelihood of its success (5;15). These elements were found to be useful when discussing case studies during the APF:

- Why: identifying the purpose and articulating the benefits, as well as the potential challenges, of collaboration. Collaborative efforts must align with the core business of all partners while at the same time striving to achieve shared objectives. In HTA, this may translate to the sharing of knowledge, skills, ideas and methodologies; reducing duplication of effort resulting in efficiency gains; delivering cost-savings, with the possibility of redirecting resources to other areas of need; and facilitating improved outcomes for patients either through equity of access or faster access to health technologies (15;17;18).
- What: defining the “problem” that needs to be solved. Agreement on the definition of the project may prevent “scope creep,” and affirm the interdependence of partners. Smaller, well-defined problems with concrete, measurable goals may be more likely to be successful (10).
- Who: are the right stakeholders/partners engaged in the collaboration? The right mix of partners needed to drive the collaboration must be identified. A landscape analysis may help identify critical stakeholders and committed partners, without whom the project may struggle to move forward or gain legitimacy. Potential partners need to have the right attributes, abilities, networks, and skills that will complement other members of the collaboration and bring strength to the project (19). There is no “recipe” for the ideal collaboration and the nature of the problem to be solved often defines the membership, structure, and process of the collaboration. Importantly, collaborations need leaders as well as champions, who can act as “collaborative capacity builders,” building and sustaining support for the partnership both within their own organizations and with all collaborative partners (10).
- How: does the collaboration have appropriate governance structures in place? Collaborations formed to achieve simple goals may need only an informal, loosely defined structure, such as a committee, requiring a time-limited investment of resources. More complex collaborations formed to address “wicked” problems, such as COVID-19, may require a more formal structure due to the number and diversity of participants, and the expected increase in the investment of time, resources, funding, and expertise. Complex collaborations need to explicitly define and articulate roles, responsibilities, and processes for all participants (15). It is important that the governance structure clearly articulates roles, responsibilities, and resources (4), including norms of operation or by-laws, mission statements, and memoranda of understandings, as well as expected outcomes and how these would be measured (10;15).
- Impact: how do we assess or measure the collaboration’s success or impact? Evaluation should measure the impact of (cumulative) actions on the intended goal(s) of the collaboration; however, it can be challenging to identify and measure outcomes directly attributable to actions (sometimes multiple actions) despite setting clear metrics for success at the outset of the project. Measuring outcomes requires capacity, technical skills and expertise, resources, and, importantly, time. Measurement criteria must be objective and evidence-based to avoid any potential conflict of interest, especially if the collaboration is politically driven or financed by vested interests (15).

Several case studies were presented in the background paper, as well as during the APF plenaries and panel sessions, including examples of within-country, regional, international, and stakeholder-driven collaborations. The many formal and informal collaborative efforts in the region demonstrate the altruistic nature of HTA organizations and the willingness to build capacity in countries currently lacking HTA capability.

### Case study 1: Thailand – Health Intervention and Technology Assessment Program (HITAP) and HTAsiaLink

Thailand’s HTA agency, HITAP, embodies this ethos by being involved in multilateral collaborative projects around the region, focusing on HTA fundamentals, building and bridging capacity gaps with technical training and workshops, and knowledge management by making learning materials and research output freely available. One of the most important collaborative projects that HITAP is currently supporting is the regional HTA harmonization work being conducted under the auspices of the Association of Southeast Asian Nations (ASEAN) alongside partners, the Philippines and Malaysia. HITAP is also one of the founding members of HTAsiaLink, with the rationale for the regional network summarized using the key elements discussed above:

- WHY – *Benefits*: build HTA capacity, reduce duplication, share HTA resources, training and knowledge across countries in Asia. *Challenges*: funding and the provision of resources (both financial and staff) by one agency to host the network. Unequal participation may be a challenge; however, all members acknowledge that participating partners are at different points on their HTA journey.
- WHAT – The network actively encourages joint research projects and the cross-country sharing of information, knowledge, and assessments.
- WHO – any HTA agency in the Asia region, young researchers, and HTA practitioners, but not open to industry participation.
- HOW – organizational structure, governance, and activities are aligned with members’ values, promoting trust. Participation is voluntary, with flexible management and no fees.
- IMPACT – increased HTA capacity across the region.

Many countries in the Asia region are experiencing similar challenges that exert pressure on healthcare systems, including an ageing population and a shift from acute to chronic, noncommunicable diseases. Evidence-informed priority setting using HTA is urgently needed to address this imbalance between health needs and financial resource constraints (20) As many countries in the region have limited capacity to conduct HTA, a regional collaboration became an attractive option (21).

Established in 2011, HTAsiaLink is one of the largest collaborative networks in the region, with a current membership of 55 agencies from 20 countries. HTAsiaLink’s core objectives include strengthening collaboration, sharing technical and methodological know-how, building HTA capacity in the region, and promoting the use of HTA in healthcare policy and decision-making across Asia (20;21). As discussed by the Breakout Groups, a major benefit and cornerstone of this regional collaboration is the close personal relationships that develop, with a foundation based on sharing similar cultural attributes, language, and common policy challenges, which not only encourages a willingness to collaborate but also builds mutual trust, respect, and open communication.

Another pillar of HTAsiaLink is the encouragement and support they offer for members to participate in joint research projects across the region that are especially relevant to the Asian setting and population, with recent projects including:

- The REALISE project to develop a framework for the use of real-world data and evidence to support decision-making in Asia (22).
- The development of an Asian-specific preference-based measure (PBM), which unlike PBMsbased on Western countries, includes dimensions and scores that reflect important health-related quality of life and preferences of East and Southeast Asians (23).
- The HTA Guideline Project aims to provide stepwise practical guidance and recommendations for lower-middle income countries to develop or update national HTA methods and/or guidelines, with a focus on HTA for benefit package design (24).

*HITAP’s take-home message? The pursuit of networks and collaborations should not be a means to an end but a strategy that aims to develop sustainable human capital and empower individuals to become HTA champions – in short, building capacity to help others build more capacity.*

As a principle, for collaboration to be successful, all relevant stakeholders should be and need to be included. There is much literature describing industry-academia collaborations (e.g., a university conducting research for uptake in innovation by industry players, or joint research projects) or even university-industry-government research collaborations. However, a major deficit identified in the background paper was the lack of literature describing industry-HTA agency collaboration, which may be due to the lack of an enabling environment and trust between the players, with HTA wanting to keep industry at “arm’s length.” One project in the region that is attempting to bridge this divide is the recently established HealthTech Hubs (Hubs) in Malaysia.

### Case study 2: Malaysian HealthTech Hubs

The Hubs offer a good example of a within-country, multi-stakeholder collaboration among government, industry, and HTA, and its collaborative rationale can be summarized as follows:

- WHY – *Benefits*: accelerated market access for innovative technologies. *Challenges*: choosing which technologies to fund.
- WHAT – A multi-partner collaboration among government, industry, and HTA
- WHO – open to local Malaysian HealthTech innovators.
- HOW – by bringing together many stakeholders under one umbrella to enable a “one-stop-shop” for innovation.
- IMPACT – increased commercialization of local innovations.

Governments often want to foster innovation to address specific healthcare needs, but there are many challenges in the early introduction of innovative technologies, not the least the lack of good-quality safety and effectiveness evidence, as well as a lack of funding for appropriate clinical studies capable of generating this evidence. To address this mismatch in expectations, where innovative technologies are not quite ready for “prime time” commercialization, Malaysia has developed a HealthTech Hub (Hub) initiative, as part of the National Technology Innovation Sandbox project (25). The Hubs are intended to allow researchers, innovators, and entrepreneurs to test their products and services in a safe and regulated “live” environment and to qualify for grants to bring those products and services to market. As the national HTA agency, the Malaysian Health Technology Assessment Section, MaHTAS, acts as the cosecretariat, with other stakeholders and partners, including hospitals that act as sandboxes (25), regulators (e.g., Medical Device Authority, National Pharmaceutical Regulatory Agency), technology manufacturers or innovators, clinicians, research institutes, and funders. By facilitating some of the regulatory requirements, the Hubs can accelerate the development of innovation from the research stage to being commercially ready, prioritizing technologies according to healthcare needs and advice provided by MaHTAS on appropriate evidence generation. As such, the Hubs act as a “single door” for the health technology industry, fostering an environment conducive to growth and innovation for health tech startups, noting that one in four applications to the Sandbox program is medical and health-related. There are currently ten science and technology priority areas, including bioscience technology, 4D/5D printing, advanced intelligent systems, sensor technology, augmented analytics, and neurotechnology. As well as providing funding opportunities, the Hubs aim to bring the key players together to enable local Malaysian companies to accelerate the commercialization of local innovations. The Hubs also collaborate with other Ministries (e.g., International Trade and Industry and Science, Technology and Innovation) to identify the needs and key priorities/challenges of the health service and, in so doing, inform areas for future research and development and investment.

As of February 2025, there are five established Hubs that are focused on medical devices designed to address an unmet clinical need previously identified by demand signaling in the health system, with another two hubs in development, one of which will be specifically tasked with identifying rehabilitation health technologies.

Collaboration occurs throughout the process with oversight from several committees and government departments; however, a key collaborative partner is the Advisory Panel, consisting of members from academia, the private sector, regulators, MAHTAS, and industry. The Hub process integrates these diverse stakeholders to provide a platform for parallel consultation and assessment, to ensure a streamlined, single-door approach for local industry that is capable of fast-tracking the adoption of innovative technologies.

*The take-home message from the Hub collaboration? Conduct early dialogue with industry partners, giving sound advice, especially on how to generate the appropriate evidence required for the regulation and adoption of an innovation into the health system.*

**Figure 2.**
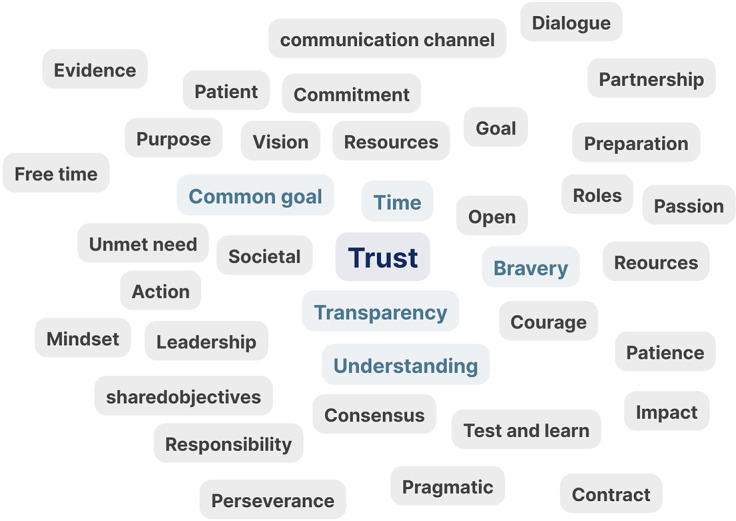
Word cloud generated by APF delegates, defining collaboration on Day 3.

Discussions both before and during the APF reiterated that collaboration is a complex, dynamic journey that requires time and effort from all partners to ensure success. Interestingly, when asked again at the end of discussions on Day-3 to define the elements of collaboration (Figure 2), delegates’ responses were similar to those on Day-1 but with the interesting addition of bravery.

What is the take-home message from the APF? Discussions emphasized the importance of finding common ground, which in HTA often centres on evidence, especially when attempting to solve “wicked” and complex problems that require multiple partners and solutions. Collaboration can, at times, be difficult to achieve, but it can reap great rewards. Above all, we must be brave, build trust and respect, and collaborate with all stakeholders to achieve better outcomes for patients and the health system.
